# Enhancing multi-spot structured illumination microscopy with fluorescence difference

**DOI:** 10.1098/rsos.171336

**Published:** 2018-03-14

**Authors:** Edward N. Ward, Frida H. Torkelsen, Robert Pal

**Affiliations:** Department of Chemistry, Durham University, South Road, Durham DH1 3LE, UK

**Keywords:** super-resolution microscopy, structured illumination, MSIM, difference microscopy

## Abstract

Structured illumination microscopy is a super-resolution technique used extensively in biological research. However, this technique is limited in the maximum possible resolution increase. Here we report the results of simulations of a novel enhanced multi-spot structured illumination technique. This method combines the super-resolution technique of difference microscopy with structured illumination deconvolution. Initial results give at minimum a 1.4-fold increase in resolution over conventional structured illumination in a low-noise environment. This new technique also has the potential to be expanded to further enhance axial resolution with three-dimensional difference microscopy. The requirement for precise pattern determination in this technique also led to the development of a new pattern estimation algorithm which proved more efficient and reliable than other methods tested.

## 1. Background

Structured illumination microscopy (SIM) and its many variants have become a staple in nanoscopic study of biological systems [1,2]. While not offering the very high resolutions of localization techniques such as photoactivation localization microscopy (PALM) [3], SIM offers biologists the ability to study living systems with a high temporal resolution. The resolution limit of fluorescence microscopy can be viewed as a limit on the maximum spatial frequency of the sample structure that can be observed. This is a result of the imaging system acting as a low-pass spatial frequency filter. The maximum observable spatial frequency is governed by the optical transfer function (OTF) of the system. Mathematically, image formation can be defined as a convolution of the sample's structure with the Fourier transform of the OTF, known as the (PSF). The PSF also represents the smallest volume to which light can be focused. In fluorescence microscopy, where a distribution of fluorophores is excited by an illumination pattern, the intensity in the image recorded is given by:

1.1 $$D(\overset{⇀}{r})=(S(\overset{⇀}{r})\cdot E(\overset{⇀}{r}))⊗\text{PSF}(\overset{⇀}{r}).$$

Here$\;D(\overset{⇀}{r})$ is the intensity at a point, $\overset{⇀}{r}$, in the image. $S(\overset{⇀}{r})$ is the sample structure and $E(\overset{⇀}{r})$ is the intensity of the excitation light at the point, $\overset{⇀}{r}$, in the sample plane. For simplicity we have assumed no magnification in image formation. ⊗ is the mathematical convolution operator and $\text{PSF}(\overset{⇀}{r})$ is the PSF of the imaging system. SIM uses interference between the structure of the sample and patterned excitation to extract higher spatial frequency information and subsequently increase resolution. The origin of the super-resolution information can be best understood by considering image formation in frequency space. Taking the Fourier transform of equation (1.1) gives

1.2 $$\tilde{D}(\overset{⇀}{k})=\tilde{S}(\overset{⇀}{k})⊗\tilde{E}(\overset{⇀}{k})\cdot \text{OTF}(\overset{⇀}{k}).$$

The tildes denote the Fourier transform of the functions and the convolution and multiplication have been switched according to the definition of the convolution operation. In the simplest case of SIM, the excitation pattern used is a striped pattern defined by

1.3 $$E(\overset{⇀}{r})=\cos({\overset{⇀}{k}}_{0}\cdot \overset{⇀}{r}),$$

where ${\overset{⇀}{k}}_{0}$ describes the direction and frequency of the pattern. Substituting into equation (1.2) and omitting constant factors yields

1.4 $$\tilde{D}(\overset{⇀}{k})\text{ = [ }\tilde{S}(\overset{⇀}{k})\text{\textbackslash{}; + }\tilde{S}(\overset{⇀}{k}\text{ + }{\overset{⇀}{k}}_{0})+\tilde{S}(\overset{⇀}{k}-{\overset{⇀}{k}}_{0})]\cdot \text{OTF}(\overset{⇀}{k}).$$

Looking at equation (1.4), one can see that the Fourier transform of the image acquired contains spatial frequency information of the sample that has been shifted by $\pm {\overset{⇀}{k}}_{0}$. This means that some of the high spatial frequency information, which would otherwise be lost, has been moved into the pass-band of the OTF. In order to extract this extra information it is necessary to capture three images of the sample, each with a shift in the phase of stripe pattern. As spatial frequencies are only shifted in the direction of ${\overset{⇀}{k}}_{0}$, to achieve isotropic resolution improvement the process needs to be repeated at different orientations of excitation pattern. Importantly, the resolution improvement possible is proportional to the spatial frequency of the excitation pattern ${\overset{⇀}{k}}_{0}$. In classical SIM, since the excitation pattern is itself diffraction-limited, the maximum possible frequency of ${\overset{⇀}{k}}_{0}$ is the cut-off frequency of the OTF, ${\overset{⇀}{k}}_{c}$. From equation (1.4) this means the highest frequency within the pass-band of the OTF is $2{\overset{⇀}{k}}_{c}$. This leads to—at best—a doubling in resolution.

Developed as a parallelized version of image scanning microscopy [3–5], an alternative to striped patterns is multi-spot SIM (MSIM), which uses a regular grid of spots (a two-dimensional delta-comb) as the excitation pattern [6]. The spots used in the pattern are diffraction-limited PSFs which, by definition, contain all spatial frequencies permitted by the OTF. It is these spatial frequencies in the spots that shift the spatial frequencies of the sample into the range of the OTF. Although this requires substantially more images to achieve even illumination, it has the advantage of being much simpler to implement on existing widefield microscopes. This is because it requires only a spatial light modulator (SLM), rather than a mechanically moving diffraction grating. Furthermore, it requires no system adjustment to account for different excitation wavelengths. Depth penetration can also be improved over striped pattern alternatives, as pinholing techniques can be applied to remove out-of-focus noise or it can be combined with multi-photon excitation [7]. In MSIM, the achievable gain in resolution is still limited by the maximum spatial frequencies in the excitation pattern, i.e. the width of the excitation PSF. As in striped-pattern SIM, the diffraction-limited nature of the excitation pattern still limits the technique to a doubling of resolution. In practical imaging conditions, however, the resolution of MSIM is often lower than that of striped-pattern alternatives due to the relative intensity of the frequency components in the PSFs. Striped patterns, containing only the maximum possible frequency ${\overset{⇀}{k}}_{c}$, concentrate the shifted spatial frequencies of the pattern to only those responsible for the maximum increase in resolution. In MSIM, where the excitation PSFs contain all permissible spatial frequencies, the highest theoretically resolvable frequencies can be drowned out by lower frequency components.

In SIM, to improve on the doubling of resolution, higher spatial frequencies must be introduced into the excitation pattern. In striped SIM this can be achieved by saturating the fluorescence as in saturated-SIM (SSIM) [8]. While increasing resolution, this has the disadvantage of greatly increasing phototoxicity and acquisition time. In MSIM, using a PSF that has been artificially tightened would introduce the necessary higher spatial frequencies into the excitation pattern to offer similarly improved resolution. Extracting the SIM data from a PSF reduced with stimulated emission depletion (STED) has been previously demonstrated, giving a 1.25 times resolution increase over STED alone [9]. While successful, this modest gain was outweighed by the cost of computational time and resources, low temporal resolution, and the high laser powers required for STED imaging. We reasoned that temporal resolution would be increased if the process were parallelized using a grid of excitation foci while using difference microscopy to simulate STED excitation [10,11]. Difference microscopy attempts to mimic STED microscopy by looking at the difference between images of the sample acquired with different illumination PSFs. By sequentially acquiring stacks of images under illumination by grids of Gaussian, then doughnut PSFs, the difference between the slices would imitate illumination by grids of sub-diffraction spots.

Using this *enhanced* MSIM (eMSIM), it would be possible to achieve a higher resolution than standalone MSIM, while maintaining compatibility with a wider range of fluorophores. Avoiding the use of a high-powered depletion/saturation beam, this technique would also exhibit lower phototoxicity than STED or SSIM imaging. Furthermore, given that eMSIM requires twice the number of images as conventional MSIM we expect imaging times to approximately double giving speeds of greater than 0.5 Hz, still suitable for live-cell imaging [6]. In this paper, we present the results of simulations of eMSIM in comparison to conventional diffraction-limited and MSIM imaging. We also propose a simple method for generating the excitation patterns, and we test a new pattern estimation algorithm to improve on existing techniques.

## 2. Methods

### 2.1. Test data

The technique was tested on two different resolution targets. A Siemens star, and a general target with resolution bars and point emitters. The simulated raw data were generated by multiplication of the test target with the appropriate excitation pattern, followed by convolution with the detection PSF. PSFs were calculated using the freely available STED3D software package for Matlab [12]. The physical parameters used were 488 nm excitation wavelength and an objective numerical aperture of 0.14 with final pixel sizes of 16 nm after deconvolution. Image intensities were normalized to unity before deconvolution. The detection PSF was generated for 519 nm emission wavelength, corresponding to the common fluorophore AlexaFluor 488. The doughnut PSFs were generated assuming a 0−2*π* phase shift applied to a right-handed circularly polarized beam as this is simple to generate in the proposed physical set-up. This gave arrays of doughnut PSFs with a full-width half-minima of approximately 128 nm. Gaussian noise was added to simulate camera readout noise. The separation of spots was chosen as approximately 450 nm as this was the closest possible without excitation overlap in the doughnut patterns. This required 14 pattern shifts in the horizontal and vertical directions. A simple weighted subtraction of the form *I*_diff_ = *I*_Gauss_ − *αI*_Dnut_ where *α* is a weighting constant was used to generate the emission difference images. The value of *α* was taken as 0.6 throughout these simulations. This was chosen as it has previously been demonstrated that for difference microscopy this minimizes the aberrations induced by misalignments and distortion in the PSFs [13].

### 2.2. Pattern estimation

Simulated post-acquisition pattern estimation was used to determine the excitation patterns. This method is generally preferable to using *a priori* knowledge of pre-calibrated patterns as it is less susceptible to aberrations arising from changes in the optical properties of different samples such as differing refractive indices. Post-acquisition estimation typically involves computationally intensive programs, and can often be unstable given underlying sample structures. For example Fourier-based methods are known to perform poorly on samples with periodic repeating structures, like the resolution bar target used [14]. We have developed what we believe to be a new estimation approach based on cross-correlation and error minimization. The raw data are first filtered using a local maxima finder. This reduces the impact of noise on the image and in tests on real-world data improves performance in samples with out-of-focus blur. Pattern spacing is determined by shifting then cross-correlating the filtered images with themselves and averaging. The rotation and is then calculated by translating and rotating a complete model pattern over the raw images and again averaging. Pattern shifts are then optimized while constraining overall even illumination. By avoiding calculations in frequency space—and therefore negating issues with repeating sample structures—this new technique is suitable for a wider range of sample structures.

### 2.3. Super-resolution reconstruction

To extract the SIM information, a combination of pattern-illuminated Fourier-ptychography (PIFP) and joint Richardson–Lucy (jRL) deconvolution was used. This combination approach was chosen as it has been shown to be the most stable method with noisy images and on a range of sample structures [15]. As in previous studies we found optimal results for approximately 5 jRL iterations and considerably more (approx. 50) PIFP iterations [14]. All simulations were performed in Matlab and accelerated with graphical processing unit (GPU) processing.

## 3. Results

### 3.1. Siemens star

**Figure 1.**
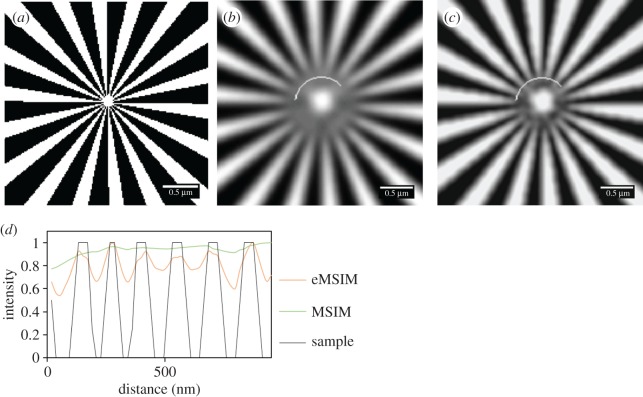
Comparison of simulated MSIM and eMSIM imaging of a Siemens star resolution target with 488 nm excitation. (*a*) Resolution target. (*b*) Simulated MSIM reconstruction. (*c*) Simulated eMSIM reconstruction. (*d*) Intensity plot along the line indicated on images. This used with 3 jRL iterations and 50 PIFP iterations.

Figure 1 shows a comparison of resolutions on a Siemens star target. Using the pattern estimation program we have compared eMSIM with conventional MSIM in a low noise environment. Looking at the intensity plot on figure 1*d* it is clear that the eMSIM has been able to resolve the spokes at higher resolution, where MSIM has underperformed resulting in blurry images with less clear structural boundaries.

### 3.2. Bar and point targets

**Figure 2.**
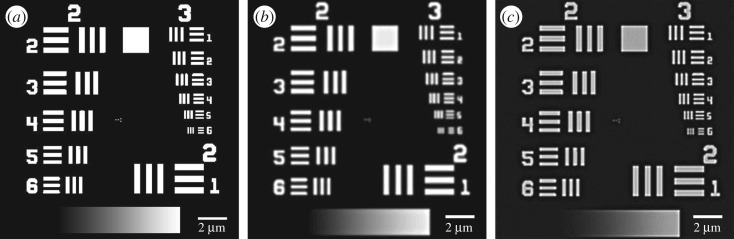
Comparison of simulated MSIM and eMSIM imaging on resolution bar target. (*a*) Resolution target. (*b*) Simulated MSIM reconstruction. (*c*) Simulated eMSIM reconstruction. This used model patterns, 3 jRL iterations and 40 PIFP iterations.

Figure 2 shows a comparison of the same techniques on a pre-defined resolution bar target. Looking at the smallest bars, eMSIM has easily resolved bars with a tighter spacing compared to MSIM.

**Figure 3.**
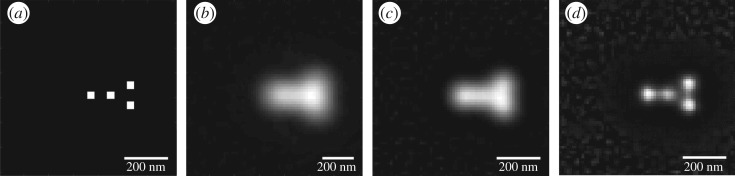
Comparison of simulated MSIM and eMSIM imaging on point emitters. (*a*) Resolution target. (*b*) Simulated diffraction-limited image with medium level noise. (*c*) Simulated MSIM reconstruction. (*d*) Simulated eMSIM reconstruction. This used our pattern estimation program, 5 jRL iterations and 10 PIFP iterations.

Figure 3 shows a comparison of diffraction-limited, MSIM and eMSIM on point emitters. Looking at the simulated point emitters, eMSIM has again offered a notable improvement in resolution over MSIM. In the ground-truth sample the sources are separated by 64 nm. As a low estimate of eMSIM resolution this represents an improvement of 1.45 times the maximum theoretically possible with MSIM.

## 4. Discussion

Our results show a clear increase in resolution over conventional MSIM microscopy even in images with simulated noise. In agreement with previous studies we found that for eMSIM and MSIM the different algorithms performed better on different targets [15]. As expected, jRL gave the strongest improvements on point and narrow bar structures although giving heightened edging artefacts, whereas PIFP reduced these artefacts but led to a significant amplification of noise in dark regions of the sample. This noise was further amplified in eMSIM, apparent on figure 3*d*. It is possible that using a mixed noise model for the jRL step might further improve performance [15]. Indeed, the noise amplification became the limiting step in these simulations as in very low noise tests increasing the subtraction constant *α* to 0.8 resolved parallel bars with 32 nm separation. However, since such low noise requirements are not typically possible in live samples this probably represents an unachievable resolution due to blur from real-life timescale natural homeostatic cell movement and intracellular organelle rearrangement.

One of the main limitations of multi-spot methods over striped-pattern alternatives is that the highest spatial frequencies shifted into the observable region are weaker when pointillistic methods. This becomes especially apparent in images with a low signal-to-noise ratio. One possible solution to this is to use a Fourier reweighting procedure before deconvolution to account for the suppression of the higher frequencies. For simplicity this reweighting was not applied during these simulations and it is possible that by doing so, the performance could be boosted to further improve the images.

Although the simulated MSIM results agree well with experimental ones in three-dimensional samples—suggesting this is a realistic model of real-world imaging—it is important to highlight the limitations of these results. The simulations are exclusively two-dimensional and it is yet unclear how the axial shape of the excitation PSFs will affect reconstruction in three-dimensional samples. Difference microscopy is typically performed on scanning confocal systems where out-of-focus light is physically rejected before the detector by the pinhole. We are currently working on extending these simulations to model three-dimensional data. Since three-dimensional FED—using axial PSF engineering—has previously been demonstrated, we hope that applying three-dimensional subtraction to eMSIM will achieve isotropic super-resolution with difference microscopy [14,15].

### 4.1. Proposed pattern generation method

**Figure 4.**
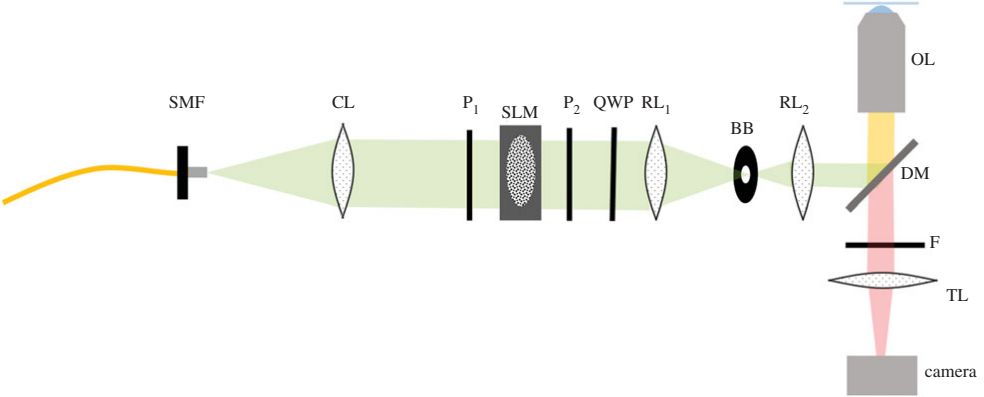
Proposed holographic projection set-up. SMF, single-mode fibre. CL, collimating lens. P1 & P2 linear polarizers. SLM, liquid-crystal transmission SLM. QWP, quarter-waveplate to generate CPL. RL1 & RL2, relay lens to image SLM onto back of objective. BB, beam block to exclude light not phase-shifted by SLM. DM, dichroic mirror. OL, objective lens. F, filter to exclude excitation light. TL, tube lens focuses image onto the camera.

Given the sparsity of the spot patterns, generating the pattern using an amplitude-only SLM in the image plane, as previously reported, rejects a significant quantity of the available light [6]. The alternative is to use a micro-lens array and modified spinning disc microscope, although this greatly increases system complexity [16]. We propose the use of holographic projection where a phase-only SLM is imaged onto the back focal plane of the objective [16,17]. Figure 4 shows the layout of a typical holographic set-up with a transmission liquid-crystal SLM. By imparting a pre-calculated phase profile on the beam, it is possible to create any desired light intensity profile in the focal plane. While the efficiency of the calculated holograms and phase-only SLMs is not yet at 100%, it still represents an order of magnitude improvement over amplitude-only SLMs. Holographic projection also negates any need for mechanically moving parts and allows for easy and dynamic manipulation of the shape of the excitation PSFs as the required phase profiles for PSF engineering are well defined [18]. This allows for the exploration of a wide range of potential excitation patterns like those required for three-dimensional eMSIM. One of the other advantages of holography is that there is very little error in the location of the excitation spots which is key for the eMSIM method. This is in comparison to the use of amplitude-only SLMs where image relay of the SLM often warps the projected image significantly and can be difficult to compensate for without pre-calibration [19]. The issue with holographic projection has typically come from ‘ghost’ spots which can appear in the pattern as well as non-uniformity in spot brightness. However, advances in computer calculation of the required phase patterns can now offer an intensity uniformity approaching 99% and developing techniques have helped to boost hologram efficiency by reducing ghost spot problems [20]. Both of these advances we believe make holographic projection the best method for generating the necessary patterns.

## 5. Conclusion

We present the simulated results of a novel structured illumination technique using PSF engineering to obtain improved resolution. While the new technique would be to some extent limited by longer acquisition times, total imaging times are still comparable to scanning methods and SSIM while using reduced illumination intensities. We believe that this new approach offers the potential for isotropic super-resolution [21,22] and propose a method that is both cost-effective and easy to implement on any existing widefield microscope.

## Data accessibility

All the data and code necessary to generate the results along with the pattern estimation program are available at: https://github.com/edward-n-ward/eMSIM.
